# The Form of N Supply Determines Plant Growth Promotion by P-Solubilizing Microorganisms in Maize

**DOI:** 10.3390/microorganisms7020038

**Published:** 2019-01-29

**Authors:** Isaac Kwadwo Mpanga, Peteh Mehdi Nkebiwe, Mira Kuhlmann, Vincenza Cozzolino, Alessandro Piccolo, Jörg Geistlinger, Nils Berger, Uwe Ludewig, Günter Neumann

**Affiliations:** 1 Institute of Crop Science (340h), Universität Hohenheim, Fruwirthstraße 20, 70593 Stuttgart, Germany; Mehdi.Nkebiwe@uni-hohenheim.de (P.M.N.); mirakulix83@gmx.de (M.K.); u.ludewig@uni-hohenheim.de (U.L.); gd.neumann@t-online.de (G.N.); 2 Department of Agriculture, University of Naples Federico II, 80138 Napoli, Italy; vincenza.cozzolino@unina.it (V.C.); alpiccol@unina.it (A.P.); 3 Institute of Bioanalytical Sciences, Anhalt University of Applied Sciences, 06406 Bernburg, Germany; joerg.geistlinger@hs-anhalt.de; 4 Eurochem Agro GmbH, 68165 Mannheim, Germany; nils.berger@eurochemgroup.com

**Keywords:** Plant Growth-Promoting Microorganisms (PGPM), P-Solubilizing Microorganisms (PSM), maize, nitrogen, stabilized ammonium, N-form, 3,4-dimethylpyrazole-phosphate (DMPP), phosphate mobilization

## Abstract

Phosphate-(P)-solubilizing microorganisms (PSM) are important drivers of P cycling in natural and agro-ecosystems. Their use as plant inoculants to improve P acquisition of crops has been investigated for decades. However, limited reproducibility of the expected effects, particularly under field conditions, remains a major challenge. This study demonstrates that the form of nitrogen fertilization has a significant impact on the performance of various fungal and bacterial PSM inoculants in maize grown on neutral to alkaline soils with limited P availability. Under these conditions, a high soil pH-buffering capacity frequently limits the efficiency of nutrient mobilization, mediated by plant roots and microorganisms via rhizosphere acidification. In a soil pH range between 7.0 and 8.0, nitrate fertilization promoting rhizosphere alkalinisation further aggravates this problem. Accordingly, in greenhouse experiments, six strains of *Pseudomonas*, *Bacillus*, *Paenibacillus*, *Streptomyces*, and *Penicillium* with proven P-solubilizing potential, completely failed to promote P acquisition in maize grown on a calcareous Loess sub-soil pH 7.6 with nitrate fertilization and rock phosphate (Rock-P) as a sparingly soluble P source. However, after replacement of nitrate fertilization by ammonium, stabilized with the nitrification inhibitor 3,4-dimethylpyrazole-phosphate (DMPP), five out of seven investigated PSM inoculants (comprising 12 fungal and bacterial PSM strains) exerted beneficial effects on plant growth and reached up to 88% of the shoot biomass production of a control supplied with soluble triple-superphosphate (TSP). Stabilized ammonium combined with PSM-inoculants improved P acquisition (*Trichoderma harzianum* T22, *Pseudomonas* sp. DMSZ 13134), while other strains particularly stimulated root growth (*T. harzianum* OMG16, *Bacillus amyloliquefaciens* FZB42), which promoted the acquisition also of other mineral nutrients, such as N, K, and Mn. A similar effect was recorded under field conditions on an alkaline clay-loam soil pH 8.6. The combination of stabilized ammonium with a range of consortium products based on *T. harzianum* OMG16, *B. amyloliquefaciens*, micronutrients, and humic acids completely compensated the effect of a TSP fertilization on field establishment, nutrient acquisition, and yield formation in maize, while non-stabilized urea-di-ammonium phosphate fertilization was largely ineffective. These findings suggest that the efficiency of PSM-plant interactions can be influenced by the form of N fertilization, offering promising perspectives for synergistic effects with stabilized ammonium fertilizers.

## 1. Introduction

Phosphorous (P) is the least soluble and consequently the least bio-available soil macronutrient, for higher plants. It is taken up by plant roots exclusively in the form of soluble mono- and divalent phosphate anions (P_i_) in the soil solution. Due to a high fixation potential in the form of Fe and Al oxides/hydroxides and the formation of sparingly soluble Fe-, Al-P at soil pH levels < 6, or Ca-phosphates at pH 7–8, soluble and easily plant available soil phosphates usually comprise less than 0.1% of the total soil P [1]. Even in well-fertilized agricultural soils, the P_i_ concentrations in the rhizosphere soil solution hardly exceed 10 µM due to rapid fixation and root uptake [2]. Theoretical considerations on plant demands suggest that the respective equilibrium concentrations in the rhizosphere soil solution needs to be replaced 20–50 times per day to meet the plant P requirements. This is not possible due to the slow diffusion-mediated desorption of sparingly soluble soil P forms [3]. Accordingly, soil-grown plants are generally facing at least latent P limitation and are largely dependent on the expression of adaptive strategies to improve P acquisition. Stimulation of root growth and fine root structures, as well as mycorrhizal associations, support the spatial acquisition of soluble P_i_. Root-induced changes in rhizosphere pH and the release of organic metal chelators can increase the solubility of immobilized soil P forms. Root-secretory phosphohydrolases can mediate the liberation of P_i_ sequestered in soil organic matter, which can comprise up to 80% of the total soil P [4]. These adaptations exhibit a large genotypic variation within plant species and cultivars. However, highly efficient P acquisition is not a widespread feature in most crops [4,5]. Accordingly, P use efficiency in agricultural production systems hardly exceeds 30% [6]. Moreover, high fixation of fertilizer P in soils and low P acquisition efficiency of plant roots are factors provoking P over-fertilization to maintain yield stability. This is associated with a high risk of irreversible P losses by surface run-off, eutrophication of surface waters, and wasting of P as a limited natural resource.

Soil microorganisms are important drivers of P turn over in soils, determining soil fertility and P availability for plants. Between 10-50 % of soil bacteria and 0.1–0.5 % of soil fungi are classified as P-solubilizing microorganisms (PSMs). They can mediate P mineralization, but also promote the solubilization of sparingly soluble inorganic P forms and even weathering of rocks and stones [6,7,8]. Similar to plant roots, PSMs are able to secrete phosphohydrolases, protons, organic metal chelators, and even mineral acids with proven potential to mineralize and solubilize the various P forms in soils [6]. Particularly in natural ecosystems, P acquisition of higher plants strongly depends on the activity of PSMs. Therefore, it is not surprising that recruitment of PSMs for symbiotic interactions is a widespread feature of plants in natural ecosystems and an important component of the adaptive plant strategies for P acquisition. Fungal PSMs are mainly found in ectomycorrhizal associations, while arbuscular mycorrhizae preferentially contribute to an improved spatial P acquisition of the host plants [4]. Similarly, many bacterial PSMs exhibit a high abundance in the rhizosphere of higher plants [6].

In face of the obvious importance of PSMs for P acquisition of higher plants, the concept to select highly efficient PSM strains as inoculants for improved P acquisition of crops has a long history dating back to the 1950s [9]. This is still promoted in numerous literature reviews [6,9,10,11]. However, although P-solubilizing properties of PSMs can be easily demonstrated on artificial growth media amended with sparingly soluble P sources, limited reproducibility of the expected effects under real rhizosphere conditions and particularly in field applications remains a major challenge [12]. More recent studies suggest that plant growth promotion and improved plant P acquisition cannot be regarded as a general PSM feature, and the expression of effects seems to be highly dependent on external factors. For example, the rhizosphere competence of microbial inoculants strongly depends on their survival in the soil environment, which can be influenced by interactions with the native soil microbiome and by abiotic stress factors [13,14]. However, the amount and type of fertilizer supply can also obviously play an important role: A recent meta-analysis by Schütz et al. [15], covering 171 publications, demonstrated plant growth-promoting effects of PSM inoculants mainly expressed in soils with moderate available P levels (25–35 kg P ha^−1^), while the efficiency declined at lower or higher ranges of P availability. This resembles the characteristics also of other beneficial plant-microbial interactions, such as symbiotic nitrogen fixation of Rhizobia with leguminous plants or plant interactions with arbuscular mycorrhizal fungi. Preferential performance of PSMs in combination with nitrogen-(N)-rich, manure-based fertilizers has been repeatedly reported by [16,17,18]. Nkebiwe et al. [19] found increased root colonisation by a PSM inoculant after ammonium depot fertilization in maize, associated with root proliferation and plant growth promotion, both in lab and field experiments.

Based on these observations, we hypothesized that the efficiency of plant-PSM interactions is influenced by the form of N fertilization. A range of pre-selected bacterial and fungal inoculants with documented P-solubilizing potential [20] was investigated in a series of pot and field experiments on soils with low P availability. Maize was selected as a host plant with a low inherent potential for mobilization of sparingly soluble soil P forms [4,21]. Rock-phosphate was provided as a sparingly soluble P source. Nitrogen supplied in different forms is frequently used in mineral fertilizers. The supplied N forms comprised nitrate, ammonium, urea, or ammonium fertilizer, stabilized with the nitrification inhibitor, DMPP (3,4-dimethylpyrazole-phosphate).

## 2. Materials and Methods

### 2.1. Pot Experiments on Artificial Sand Sub-soil Substrates

The first and second experiments were designed on 10 June 2014 and 28 February 2015 respectively using artificial mixtures of washed quartz sand and a calcareous Loess subsoil with a high P sorption capacity, dominated by sparingly soluble Ca-P with low levels of organic matter to minimize P supply via mineralization and to ensure that plant P acquisition mainly depended on mineral P solubilization.

#### 2.1.1. Substrate Characteristics and Fertilization

Plant-available P: P_CAL_: 5 mg kg^−1^ [22]; pH_CaCl2_: 7.6; C_org_: < 0.3%; N_total_ 0.02%; CaCO_3_: 23%.

The first experiment employed a mixture of 80% soil and 20% (*w*/*w*) quartz sand (0.6–1.2 mm Ø). The substrate was fertilized by homogenous incorporation of (mg kg^−1^ substrate): N (Ca(NO_3_)_2_) = 100; P 150 (Rock phosphate, 7.6% P, Timac-Agro, Troisdorf, Germany) or Ca(H_2_PO_4_)_2_) for the positive P control); K (K_2_SO_4_) = 150; Mg (MgSO_4_) = 50; Zn (ZnSO_4_) =2.6; Cu (CuSO_4_) = 1.0; and 20 μmol Fe kg^−1^ substrate (Sequestrene138, 6% Fe). Each pot was filled with 2.9 kg of substrate and moisture was adjusted daily to 18% (w/w) = 60% substrate water holding capacity (WHC).

For the second experiment, the addition of quartz sand was increased to 70% (*w*/*w*). The Rock-P fertilization was combined with two N forms at 100 mg N kg^−1^ substrate: (1) 100% NO_3_-N as Ca(NO_3_)_2_, and (2) 80% NH_4_-N as DMPP-(3,4-dimethylpyrazole-phosphate)-stabilized (NH_4_)_2_SO_4_ (Novatec solub, Compo Expert GmbH, Münster, Germany) with 20% NO_3_-N as Ca(NO_3_)_2_). A negative control without P fertilization and a positive control with soluble Ca(H_2_PO_4_)_2_ were included as additional treatments with nitrate fertilization. For the remaining nutrients, substrate fertilization was identical with experiment 1.

#### 2.1.2. PSM Inoculation and Plant Culture

Experiment 1: *Pseudomonas* sp. DSMZ 13134, Proradix^®^, Sourcon Padena GmbH, Tübingen, Germany (Pro: 1 × 10^9^ CFU kg^−1^ substrate), *Penicillium* sp. PK 112, Biological Fertilizer OD, Bayer CropScience Biologics GmbH, Wismar, Germany (BFOD, 1 × 10^8^ spores kg^−1^ substrate), *Paenibacillus mucilaginosus*, Abitep GmbH, Berlin, Germany (Paeni, 1 × 10^9^ spores kg^−1^ substrate) and Vitalin SP11, Vitalin Pflanzengesundheit GmbH, Ober-Ramstadt, Germany (SP11, 20 mL of 0.2% suspension kg^−1^ substrate). Vitalin SP11 comprises *Bacillus subtilis*, *Pseudomonas* sp., *Streptomyces* spp., humic acids and extracts of the seaweed, *Ascophyllum nodosum*.

Experiment 2: *Pseudomonas* sp. DSMZ 13134, Proradix^®^ (Pro: 1 × 10^9^ CFU kg^−1^ substrate).

The inoculants were suspended in 2.5 mM CaSO_4_. Maize seeds (*Zea mays* L. var Colisee) were soaked for 10 min with the microbial suspensions, sown at 3 cm depth and thereafter 20 mL PSM suspension was inoculated into the seeding hole with two additional weekly applications close to the stem of the plants. Plants were arranged in a completely randomized design with 4 replicates per treatment for 41 days (experiment 1) and with 5 replicates for 36 days (experiment 2) under greenhouse conditions (air temperature range: 11–30 °C, average 21 °C) with additional light 12 h d^−1^, average light intensity: 275 µM m^−1^ s^−1^.

### 2.2. Pot Experiment on Field Soil

The experiment was established on 15 August 2015 on an organic farming field soil with moderately low P availability, freshly collected from the A horizon at the experimental station, Klein-Hohenheim, Hohenheim University, Stuttgart, Germany), to include a native top-soil microflora.

#### 2.2.1. Substrate Characteristics and Fertilization

Soil characteristics: Clay-loam, pH_CaCl2_ = 7.0; P_CAL_ = 36.7 mg P Kg^−1^; N_total_: 0.15%; C_org_: 1.28%; substrate mixed with 30% (*w*/*w*) quartz sand for improvement of soil structure.

The basal fertilization comprised (mg kg^−1^ substrate): N 100 as DMPP-stabilized (NH_4_)_2_SO_4_ or Ca(NO_3_)_2_, P 100 (Rock-P or Ca(H_2_PO_4_)_2_ for the positive control); K 150 (as K_2_SO_4_) and Mg 50 (as MgSO_4_). No micronutrient fertilisation was performed in this experiment. Each pot was filled with 3 kg of substrate and moisture was adjusted daily to 21% (*w*/*w*) = 60% substrate water holding capacity (WHC).

#### 2.2.2. PSM Inoculation and Plant Culture

Seven PSM inoculants were tested in comparison with a non-inoculated control, with Rock-P as a sparingly soluble P source in combination with an N supply as DMPP-stabilized NH_4_^+^: *Pseudomonas* sp. DSMZ 13134 (Proradix); *Trichoderma harzianum* T22 (Trianum-P, Koppert, Biological Systems, Berkelen Rodenrijs, The Netherlands); *Penicillium* sp. PK 112 (BFOD), *Paenibacillus mucilaginosus* (Paeni), *Bacillus amyloliquefaciens* FZB42 Rhizovital42^®^ (Abitep GmbH, Berlin, Germany); Vitalin SP11, and CombiFectorA: *Trichoderma harzianum* OMG16 + Vitabac with five *Bacillus* strains (Bactiva GmbH, Straelen, Germany) + Zn/Mn, Institute of Bioanalytical Sciences, Bernburg, Germany). Furthermore, the best performing PSM strain, Proradix [20], was tested also with Rock-P and nitrate-based fertilization, as a reproduction of experiment 2, described under Section 2.1. Two additional non-inoculated treatments included an unfertilized control and a positive control, supplied with soluble triple-superphosphate (TSP, 100 mg P kg^−1^ substrate) and Ca(NO_3_)_2_ fertilization (100 mg N kg^−1^ substrate). Inoculation was performed as described under Section 2.1.2. Plants were arranged in a completely randomized design with five replicates per treatment for 35 days under greenhouse conditions (air temperature range: 13–32°C, average 20 °C) with additional light 12 h d^−1^; average light intensity: 275 µM m^−1^ s^−1^.

### 2.3. Field Experiment

The field trial was conducted in 2016 at the “Experimental Station of the Department of Agriculture of Napoli Federico II”, located at Castel Volturno, in an agricultural area 60 km north of Naples, (CE) Campania, Italy; annual mean temperature: 15.6 °C; average annual precipitation: 879 mm.

#### 2.3.1. Soil Characteristics and Fertilization

The soil was classified as clay loam (Vertic Xerofluvent), pH_H2O_ 8.6; available NaHCO_3_-extractable P_Olsen_ 11 mg kg^−1^ N_total_: 0.13%, C_org_ 1.5%. Nitrogen and phosphate fertilization was performed (1) according to the local farmers’ practice (urea = 180 kg N ha^−1^ and non-stabilized di-ammonium phosphate (DAP) = 50 kg P ha^−1^); (2) as a negative control with DMPP-stabilized ammonium sulfate (NovaTec 21 solub = 150 Kg N ha^−1^) without additional P fertilization; (3) as a positive control with DMPP-stabilized ammonium sulfate (150 Kg N ha^−1^) and triple superphosphate (TSP= 50 kg P ha^−1^) and (4) combinations of DMPP-stabilized ammonium sulfate (150 Kg N ha^−1^) with selected PSM-inoculants, but without additional P fertilization.

#### 2.3.2. PSM Inoculation and Plant Culture

PSM products comprised: (1) Combifector A (a combination product of *Trichoderma harzianum* OMG16 + Vitabac = 5 *Bacillus* strains and micronutrients Zn/Mn, Institute of Bioanalytical Sciences, Bernburg, Germany; (2) Combifector B (a combination product of *Trichoderma harzianum* OMG16, + *Bacillus amyloliquefaciens* (RhizoVital FZB42) and micronutrients Mn/Zn, Institute of Bioanalytical Sciences, Bernburg, Germany, ABiTEP GmbH, Berlin, Germany; (3) *Bacillus amyloliquefaciens*, Rhizovital FZB42^®^ ABiTEP GmbH, Berlin, Germany + humic acids from composted cow manure produced on a farm in Castel Volturno; and (4) a seaweed extract—*Bacillus amiloquefaciens* seed dressing formulation provided by Group Limagrain, Saint-Beauzire, France). Combifector A and B (1+2), were applied at sowing by broadcast top-soil incorporation at a dosage of 100 g ha^−1^, equivalent to 1 × 10^12^ fungal spores plus 1 × 10^12^ bacterial spores ha^−1^. *Bacillus amyloliquefaciens*, Rhizovital FZB42 with humic acids at a dosage of 5 kg ha^−1^ (3) was inoculated into the seeding row via band application, and the *B. amyloliquefaciens*—seaweed extract formulation (4) was provided in the form of pre-coated maize seeds.

The experimental area was divided into 40 m^2^ plots under a randomized block design with four replicates. Maize seeds (*Zea mays* L. cv 30.600, Group Limagrain, Saint-Beauzire, France) were sown at the beginning of June with a distance of about 10 cm and 75 cm inter-row distance, with a plant density of 7 plants m^−2^. Each treatment was replicated four times. Plant establishment was monitored at the V6 stage at 42 DAS by shoot biomass determination. Final grain harvest was performed in early November.

### 2.4. Plant Biomass and Root Length Determination

At final harvest, the dry biomass of the shoots was determined after 3 d of being oven-dried at 65 °C. The roots in each pot were washed out from the soil substrate and were stored in 30% (*v*/*v*) ethanol. The roots were later separated, submerged in a water film in transparent Perspex trays, and digitalized using a flat-bed scanner (Epson Expression 1000 XL, Tokyo, Japan). Subsequently, the root length of the digitalized samples was measured using the WinRHIZO root analysis system (Reagent Instruments, Quebec, QC, Canada). Thereafter, the root samples were oven-dried for 2 days at 65 °C for the determination of dry matter.

### 2.5. Shoot Mineral Analysis

For both experiments, plant mineral nutrient analysis was performed as follows: Tomato shoot N was measured with a Vario Max CN macro-elementar analyser (Elementar Analysensysteme, Hanau, Germany). For P, K, Ca, and Mg, a microwave digestion method was employed for the wet ashing of finely ground dry plant materials (250 mg) in 1 mL of deionized water, 2.5 mL concentrated HNO_3_ (1:3), and 2 mL H_2_O_2_ (30%). Digestion was performed in a microwave digestion system (Ethos, MLS, Leutkirch, Germany) for 1 h and allowed to cool for 30 min. Approximately 5 g of activated charcoal was added for sample decolouration, mixed well by shaking, and allowed to settle during 15 min. The samples were filtered with ashless MG 640d Blue ribbon filter paper (Macherey & Nagel, Düren, Germany). Phosphate was estimated spectrophotometrically (Hitachi LtD., Tokyo, Japan) according to [23]. Magnesium, calcium, zinc, and manganese were measured by atomic absorption spectrophotometry (iCE 3000 series, Thermo Fischer, Dreieich, Germany) and potassium by flame emission spectrophotometry (Eppendorf-ELEX6361, Netheler+Hinz, Hamburg, Germany).

## 3. Results

### 3.1. Experiments on Artificial Growth Substrates (Sub-Soil-Sand Mixtures)

**Figure 1:**
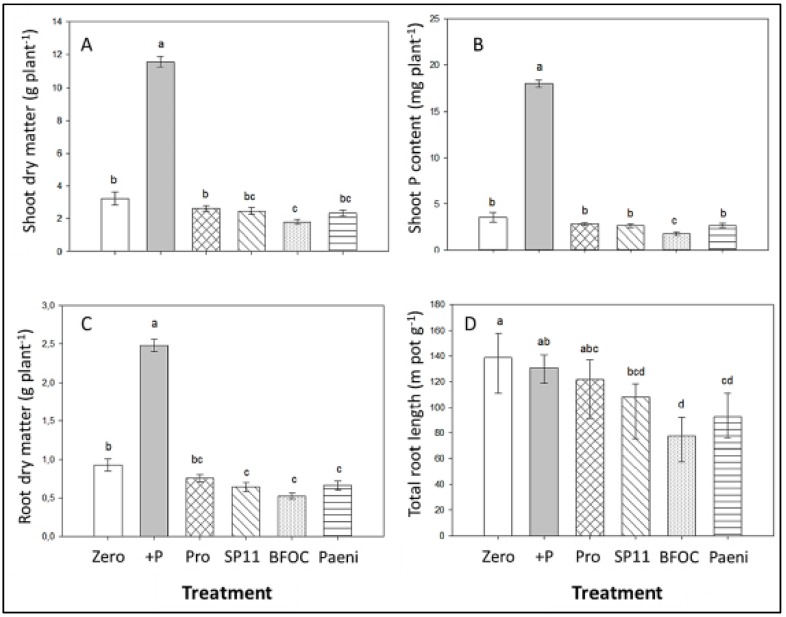
Shoot biomass (**A**), shoot P content (**B**), root dry matter (**C**), and total root length (**D**) of maize (cv Colisee) grown on a calcareous Loess subsoil (pH 7.6)—sand mixture (80/20% *w*/*w*), supplied with and without (Zero) P fertilization in the form of Rock-P (RP) or soluble Ca(H_2_PO_4_)_2_ (+P) and calcium nitrate fertilization. RP variants inoculated with *Pseudomonas* sp. DSMZ 13134 Proradix (Pro); SP11, Vitalin SP11 (SP11); *Penicillium* sp. PK 112 (BFOC); and *Paenibacillus mucilaginosus* (Paeni). Means of four replicates. One-way ANOVA, Tukey test. Different letters indicate significant differences (*p* < 0.05).

To study PSM-induced mobilization of sparingly-soluble soil P, maize (cv Colisee) was used as a test crop with low adaptive potential for root-induced P solubilisation [4]. Plants were inoculated with different PSMs of fungal and bacterial origin, comprising three single-strain inoculants (*Pseudomonas* sp. DSMZ 13134 Proradix^®^(Pro), *Paenibacillus mucilaginosus* (Paeni), *Penicillium* sp. PK 112, Biological Fertilizer OD (BFOD) and one consortium product, Vitalin SP11, with a combination of *Bacillus subtilis*, *Pseudomonas* sp., *Streptomyces* spp., humic acids, and extract of the seaweed, *Ascophyllum nodosum*. Pilot experiments revealed that the selected microbial PSMs were able to solubilize insoluble tri-calciumphosphates (Ca-P), rock phosphate (RP), and sewage sludge ash (SA), added to artificial growth media, and Proradix was identified as the most efficient PSM strain [20]. The plants were cultivated on a calcareous Loess subsoil substrate (80% soil/20% sand, pH 7.6) with low P availability (5 mg P_CAL_ kg^−1^), low organic matter content < 0.3%), and sparingly soluble rock P as the exclusive P source. This experimental setup ensured that plant P acquisition was only possible after Ca-P solubilisation. However, despite the proven P-solubilizing potential, all microbial inoculants failed to stimulate P acquisition of the test plants and even exerted inhibitory effects on plant growth in comparison with a non-inoculated control (Figure 1A,C,D). Accordingly, P shoot accumulation was not increased in the PSM-treated variants. By contrast, shoot biomass production increased by 300% and shoot P accumulation by 500% in maize plants supplied with soluble Ca(H_2_PO_4_)_2_ as a positive control (Figure 1A,B).

Based on these results, it was hypothesized that a high pH-buffering capacity of the calcareous soil substrate with 23% CaCO_3_ counteracts PSM-induced acidification of the growth medium and thereby microbial Rock-P solubilisation. To test this hypothesis, the pH buffering capacity of the growth substrate was reduced by increasing the sand content from 20 to 70% (*w*/*w*). Moreover, as an additional fertilisation treatment to the N supply via calcium nitrate, a variant with ammonium-dominated N application (80% (NH_4_)_2_SO_4_, stabilized with the nitrification inhibitor DMPP + 20 % Ca(NO_3_)_2_) was included in order to promote Rock-P solubilisation by ammonium-induced rhizosphere acidification [4]. Proradix, pre-characterized as PSM with the highest P-solubilizing potential [20], was used as an inoculant.

**Figure 2:**
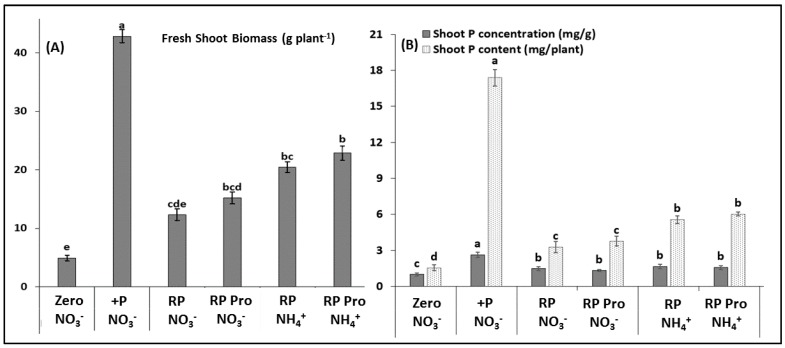
Shoot biomass (**A**), shoot P content, and concentration (**B**) of maize (cv Colisee) grown on a calcareous Loess subsoil pH 7.6—sand mixture (30/70% *w*/*w*), supplied with and without (Zero) P fertilization in the form of Rock-P (RP) or soluble Ca(H_2_PO_4_)_2_ (+P). RP variants with and without *Pseudomonas* sp. DSMZ 13134 Proradix (Pro) inoculation in combination with Ca-nitrate (NO_3_^−^) or DMPP-stabilized ammonium (NH_4_^+^) fertilization. Means of five replicates. One-way ANOVA, Tukey test. Different letters indicate significant differences (*p* < 0.05).

In the variants with nitrate fertilization and Rock-P supply, PSM inoculation had no significant effect on shoot biomass production (Figure 2A) and P accumulation (Figure 2B) of the maize plants. Biomass production reached only 35%, and P accumulation 24%, as compared with the positive TSP control supplied with soluble P. Replacement of nitrate by stabilised ammonium significantly increased shoot P accumulation (Figure 2B), but a significant increase in shoot biomass production by 92% was exclusively achieved by the combination of ammonium supply with PSM inoculation (Figure 2A). However, P shoot concentration and P accumulation of the ammonium variants with and without PSM inoculation were not significantly different. With the exception of the positive control supplied with soluble TSP, the P nutritional, status of the remaining variants was critical (< 0.3%, Campbell 2009) (Figure 2B).

### 3.2. Pot Experiment on Field Soil

Since stabilized ammonium fertilization exerted beneficial effects on the plant growth-promoting potential of the PSM strain, Proradix, on a sand-soil substrate supplied with sparingly soluble Rock-P as major P source (Figure 2), an additional experiment was conducted under more realistic conditions, using a clay-loam organic farming field soil (pH 7.0) with moderately low P availability (P_CAL_ 37 mg kg^−1^). Phosphate was supplied as Rock-P or in the form of soluble triple-superphosphate (TSP) as a positive control. To evaluate synergistic effects of PSM inoculants with stabilized ammonium fertilization, two fungal (Trianum-P = *Trichoderma harzianum* T22, BFOD = *Penicillium* sp.) and three bacterial single-strain inoculants (Proradix = *Pseudomonas* sp. DMSZ 13134; Rhizovital = *Bacillus amyloliquefaciens* FZB42; and *Paenibacillus mucilaginosus*), as well as two consortium products (SP11 and Combifector-A), pre-characterized as PSMs [20], were selected for inoculation of maize plants (cv Colisee). Proradix, characterized as the strain with the highest P-solubilizing potential [20], was investigated also in combination with nitrate fertilization.

#### 3.2.1. Shoot Growth and Root Development

**Figure 3:**
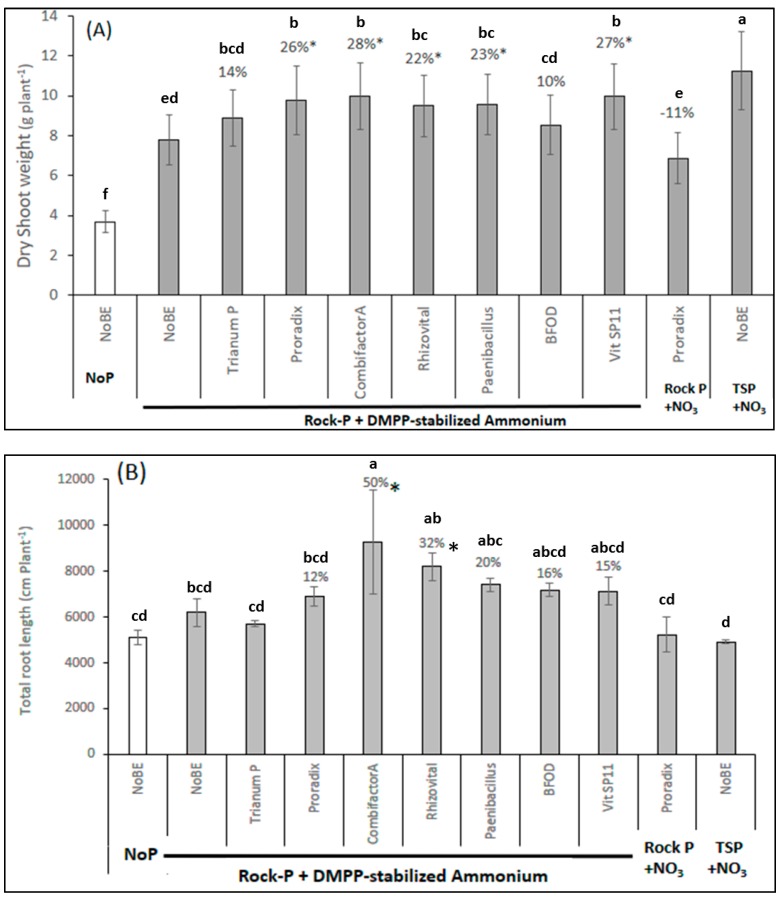
Shoot biomass (**A**) and total root length (**B**) of maize (cv Colisee) grown on a clay-loam, organic farming soil (pH 7.0), supplied with and without (No P) P fertilization in form of Rock-P or soluble triple superphosphate (TSP). N supply in the form of Ca-nitrate (NO_3_) or DMPP-stabilized ammonium. Microbial inoculants: *Trichoderma harzianum* T22 (Trianum P), *Pseudomonas* sp. DSMZ 13134 (Proradix), *Trichoderma harzianum* OMG16 + 5 *Bacillus* strains (Combifector-A); *Bacillus amyloliquefaciens* FZB42 (Rhizovital), *Paenibacillus mucilaginosus*, *Penicillium* sp. PK 112 (BFOD), Vitalin SP11 (VitSP11), or no inoculation (NoBE). Means of five replicates. One-way ANOVA, Tukey test. Different letters indicate significant differences (*p* < 0.05); * indicates significant differences after pairwise comparison of PSM-inoculated variants versus the non-inoculated control with ammonium fertilization (*t*-test, *p* < 0.05).

Analysis of shoot biomass production revealed P as the limiting nutrient, indicated by a 205% increase after soluble TSP application as compared with the unfertilized control. Stabilized ammonium with Rock-P had a fertilizer effect of 111%. Biomass production in the PSM-ammonium combinations was significantly increased in all variants compared with the non-inoculated control, with the exception of the two fungal strains, Trianum P and BFOD. Similarly, the combination of Proradix with nitrate fertilization revealed no plant growth-promoting effects. (Figure 3). Stimulation of shoot growth by PSM inoculation was associated with a clear trend for increased root length development, although the effect was significant only for the single strain inoculant, Rhizovital FZB42 (+ 32%), and the consortium product, Combifector-A (+ 50%. Figure 3B), after pairwise comparison with the non-inoculated control.

Similar beneficial growth effects of ammonium fertilization on selected PSM strains have been recorded in additional experiments on different soils with a pH range between 5.7 and 7.9 and maize, spring wheat, and tomato as target crops (summarized in Table S1).

#### 3.2.2. Mineral Nutrient Status

Concerning the plant nutrient status (Table 1), significant PSM effects were recorded for nitrogen (N), phosphate (P), potassium (K), and manganese (Mn). Magnesium and zinc concentrations were in the sufficiency range for all treatments).

The P nutritional status of the maize plants was critical (< 0.3%, [24]) in all investigated variants, even with soluble TSP fertilization. The combination of stabilized ammonium with Rock-P increased the P shoot concentration by 27% as compared with the unfertilized control, without a further increase by additional PSM inoculation. However, P shoot accumulation was significantly increased in the ammonium combinations with Trianum P and Proradix after pairwise comparison with the non-inoculated control (*t*-test, *p* = 0.05).

The N status was critical in the ammonium-Rock-P variant (26 mg g DM^−1^), but the N concentration reached the sufficiency range [24] for all tested PSM inoculants. Nitrogen shoot accumulation increased significantly in the Proradix-, Rhizovital-, SP11, and Combifector-A-ammonium combinations. The K status was sufficient in all treatments and shoot K accumulation was further increased by all PSM treatments except BFOD by pairwise comparison with the non-inoculated control. The manganese status of the plants supplied with Rock-P and stabilized ammonium was critical [24], but was significantly increased by 50% to the sufficiency range by PSM-inoculation.

### 3.3. Field Experiment

To evaluate the beneficial effects of stabilized ammonium fertilization on plant-PSM interactions in maize under practice conditions, a field experiment was established at the experimental Station Castel Volturno, in an agricultural area 60 km north of Naples, (CE) Campania, Italy, on an alkaline clay loam soil (Vertic Xerofluvent) of pH 8.6 with moderate P availability (11 mg kg^−1^ soil) according to P_Olsen_ extraction [25].

The investigated BEs comprised combination products of bacterial and fungal strains pre-tested in the pot experiments (Rhizovital = *Bacillus amyloliquefaciens* FZB42, Combifector A = Trichoderma harzianum OMG16 + Vitabac (5 *Bacillus* strains), Combifector B = *Trichoderma harzianum* OMG16 and *Bacillus amyloliquefaciens* FZB42). The microbial strains were combined with Zn/Mn (Combifector A/B), humic acids (FZB42 + HA), or seaweed extract (*B. amyloliquefaciens* + SW) and applied by soil incorporation (CombiA/B, FZB42+HA) and via seed dressing (*B. amyloliquefaciens* + SW). The treatments comprised variants without P fertilization supplied with DMPP-stabilized ammonium sulfate with or without application of microbial inoculants. A triple superphosphate combination with stabilized ammonium was included as a positive control with soluble P supply and a farmer´s practice variant with urea and non-stabilised di-ammonium phosphate fertilization was included as an additional control. An intermediate harvest during early growth of the maize plants was performed at the V6 stage (42 DAS) and final grain yield was recorded at V12.

During early growth, plant biomass production of the control without P fertilization supplied with stabilized ammonium was significantly increased by 24% after supplementation with soluble P (TSP). Replacing TSP application by PSM inoculation resulted in even stronger responses in shoot biomass production, with the largest effect induced by Combifector-B (+ 40%). Early plant growth promotion translated into a significant increase in grain yield by 0.8 t ha^−1^ (+ 5.2%) in the TSP variant, while significant yield effects in the PSM treatments were recorded for CombifectorA/B and the FZB42+HA variants, with the largest effect (1.0 t ha^−1^, + 6.5%) in the Combifector-B-ammonium combination. The farmer´s practice of fertilization with non-stabilized urea and di-ammonium phosphate had no significant effects in terms of biomass production during plant establishment and the smallest effect on final grain yield (+ 3.2%), as compared with the control supplied with stabilized ammonium without P fertilization. The P status was sufficient, and the N status was low to critical without significant treatment differences [24]. Shoot P accumulation was significantly increased by Combi-B and FZB42+HA application and also N accumulation increased particularly in response to the PSM treatments, while this effect was less expressed in the farmer´s practice and TSP variants (Table 2).

## 4. Discussion

Understanding the contribution of PSM inoculants to plant growth promotion and the best conditions for their efficient performance at a mechanistic level is a challenge. Many studies have characterized the solubilization potential of PGPMs from sparingly soluble tri-Ca-phosphate on artificial media, followed by pot and/or field experiments with inoculated host plants on P limited soils, and successful examples of plant growth promotion are frequently interpreted as a consequence of microbial P solubilization [6]. There is no doubt that soil microbial activities play an important role in P mineralization and for solubilization of sparingly soluble mineral forms of soil P. However, PSM-host plant interactions involved in plant growth promotion are obviously more complex.

### 4.1. PGPM Effects on Artificial Sand/sub-soil Substrates

Testing a range of four PSM inoculants, based on six bacterial and fungal strains with proven potential for solubilization of sparingly soluble tri-calcium phosphate [20,26,27], was performed in a culture system, based on a calcareous Loess subsoil (pH 7.6) with sparingly-soluble Ca-phosphate (Rock-P) as the sole P source. Maize was used as a crop with a low inherent potential for root-induced P solubilisation [4]. Under these conditions, plant P uptake was almost exclusively dependent on Ca-P mobilization. However, all tested inoculants with P-solubilizing potential completely failed in terms of plant growth promotion and PSM-assisted P acquisition, associated with a severely P-deficient nutritional status of the host plants (1.1 mg g^−1^ shoot DM). Normal plant development required supplementation with soluble triple-superphosphate (TSP; Figure 1). Similar results have been recently reported for experiments conducted in seven countries on soils with low-P availability and/or supply of sparingly soluble P sources, such as Rock-P, slags, and ashes in four different crops (maize, barley, wheat, tomato) with 13 PSM strains [4,17,18,28]. Based on these findings, it was hypothesized that on neutral to alkaline soils, a high pH buffering capacity might be a major factor, limiting the efficiency PSM-induced P solubilisation in the rhizosphere via the release of protons, organic, and mineral acids [6]. Similar limitations have been previously reported also for mobilization of P or Fe via root-induced rhizosphere acidification [4,29].

To test this hypothesis, the experiment was repeated with the same calcareous sub-soil, mixed with 70% (*w*/*w*) quartz sand to reduce the pH-buffering capacity of the substrate. As an additional variant, nitrate-based N fertilization was partially replaced by stabilized ammonium to promote rhizosphere acidification via proton release from plant roots and microorganisms for charge-balance of ammonium uptake [4]. Under the conditions of lower substrate buffering, already Rock-P supply tended to increase plant biomass production, but a significant effect compared with the unfertilized control (+ 207%) was recorded exclusively in combination with the *Pseudomonas* strain, DMSZ 13134 (Proradix), pre-elected as the most efficient PSM strain in pilot experiments [20]. As expected, ammonium fertilization further stimulated shoot biomass production, but again a significant effect compared with the nitrate variant (+ 130%) was recorded only in combination with Proradix (Figure 2A). Interestingly, in contrast to the effects on plant growth promotion, shoot P accumulation was significantly increased by Rock-P fertilization in the nitrate variant (+ 110%) and by ammonium versus nitrate fertilization (+ 71%), without additional effects induced by PSM inoculation. This finding suggests that PSM-induced plant growth promotion was not simply a consequence of PSM-mediated P solubilisation. Taken together, the results indicated that the pH-buffering capacity of the substrate can indeed represent a limiting factor for PSM-assisted fertilization strategies to improve plant acquisition of acid-soluble Ca-P fractions in soils. The combination with stabilized ammonium fertilizers supporting rhizosphere acidification may act as a suitable strategy to promote PSM performance. However, the effects are not necessarily related with direct promotion of the P-solubilizing potential of the PSM inoculants.

### 4.2. PGPM Effects on Field Soil

Plant growth-promoting effects of microbial inoculants can be demonstrated most easily on artificial growth substrates lacking a native soil microflora with potentially competing properties in terms of root colonization. This was of course also the case in the first experiment demonstrating positive PSM effects conducted on an artificial sand-subsoil mixture. Therefore, the experiment was repeated, using a real field soil pH 7.0 with moderately low P availability (P_CAL_ 37 mg kg^−1^) and Rock-P fertilization. Again, stabilized ammonium fertilization combined with Rock-P increased the shoot biomass compared with the unfertilized control. Shoot biomass production was further increased by combination of Proradix with stabilized ammonium, which was not the case for the combination of Proradix with nitrate fertilization (Figure 3). In this experiment, additionally, a wider range of pre-selected PSMs [20] was tested under the same conditions. The selection comprised five single-strain inoculants with two fungal (Trianum-P = *Trichoderma harzianum* T22, BFOD = *Penicillium* sp) and three bacterial strains (Proradix = *Pseudomonas* sp. DMSZ 13134; Rhizovital = *Bacillus amyloliquefaciens* FZB42; *Paenibacillus mucilaginosus*), as well as two consortium products (SP11 used in experiment 1 and Combifector-A (= *Trichoderma harzianum* OMG16 + Vitabac (five *Bacillus* strains) + Zn/Mn).

With the exception of the two fungal strains, all PSMs significantly increased shoot biomass production in combination with stabilized ammonium fertilization and reached about 88% of the biomass of the plants supplied with soluble TSP (Figure 3). This was associated with a clear trend for increased root length development with significant effects for FZB42 (+ 31%) and Combifector-A (+ 50%). By contrast, the two fungal inoculants and the nitrate-Proradix combination, ineffective in shoot growth promotion, also had no or only marginal effects on root growth. Similar to the experiment with the artificial sand-sub-soil substrate (70/30), ammonium fertilization in combination with Rock-P significantly increased the P tissue concentrations compared with the unfertilized control, but no further increase was recorded after PSM inoculation (Table 1). Shoot accumulation of P was significantly increased after inoculation with the bacterial PSM, Proradix, and the fungal PSM, Trianum-P, but the fungal strain had no effect on root growth or shoot biomass production (Figure 3, Table 2). By contrast, Combifector-A with the largest impact on root length development (+ 50%) had no significant effect on shoot P accumulation (Figure 3B, Table 2). The results indicated once more that the plant growth-promoting effects of the investigated inoculants in combination with stabilized ammonium fertilization were obviously not related to direct PSM-assisted P solubilisation in the rhizosphere. However, a closer look to the mineral nutritional status of the plants revealed that P was not the only limiting nutrient, and critical levels of N (< 30 mg g^−1^ DM) and Mn concentrations (0.02 mg g^−1^ DM) were recorded in the control treatment supplied with stabilized ammonium and Rock-P fertilization (Table 1). The microbial inoculants increased both the N and Mn nutritional status to the sufficiency range (Table 1) and a significant increase of N and Mn shoot accumulation was observed for the Proradix, Rhizovital, SP11, and Combifector-A-ammonium combinations by pairwise comparison with the non-inoculated control (Table 2), associated with increased shoot biomass production (Figure 3A).

Taken together, the results suggest a scenario of synergistic interactions between fertilizer supply and plant growth-promoting properties of the selected PSM inoculants: On neutral to alkaline soils with low P availability, crops with a low inherent potential for P solubilisation are frequently facing problems of P limitation. The inoculation with PGPMs to improve plant P acquisition is not successful in this case, since the weak P-deficient plants are not able to support efficient root colonization by the PSM inoculants and the establishment of a functional plant-microbial interaction. The fertilization with stabilized ammonium fertilizers could partially overcome this limitation by improving the P nutritional status, probably mediated by the well-documented root-induced rhizosphere acidification [4], contributing to solubilisation of Ca-phosphates. The more vital status of these plants promoted root colonisation by the microbial inoculants, which were, in turn, able to express their plant growth promoting potential. This is in line with previous reports on beneficial effects of P starter supply on the establishment of arbuscular mycorrhizal associations and the Rhizobium symbiosis in leguminous plants [30,31]. It also confirms the findings of the recent meta-analysis by Schütz et al. [15], which demonstrated that plant growth promoting effects of PSMs can be expected on soils with moderately low P availability (25–35 kg P ha^−1^), but not on low-P soils or under sufficient P supply. Under these conditions, plant growth promotion is not necessarily caused by PSM-mediated P solubilisation. Stimulation of root growth induced by the inoculants can contribute to the acquisition of other potential growth-limiting nutrients and may also promote ammonium-induced P solubilization by the development of a larger acidifying root system.

### 4.3. PGPM Effects under Field Conditions

This scenario was evaluated additionally under field conditions on an alkaline clay loam soil (Vertic Xerofluvent) pH 8.6 with a P availability (P_Olsen_: 11 mg kg^−1^ soil) considered as moderate for maize cultivation [25,32]. In face of the high soil pH and moderate P availability [25], no Rock-P fertilization was included into this experiment and the performance of microbial inoculants in combination with stabilized ammonium was compared without P fertilization versus TSP fertilization and fertilization according to the farmer’s practice, which comprised di-ammonium phosphate and urea without nitrification inhibitors. Due to the promising plant growth-promoting effects of PGPM combinations in the previous experiment (Figure 3A), a range of consortium products were tested as microbial inoculants: Combifector-A (see Section 4.2) Combifector-B (*Trichoderma harzianum* OMG16 + *Bacillus amyloliquefaciens* FZB42 + Zn/Mn), *Bacillus amyloliquefaciens* FZB42 + humic acids, and *Bacillus amyloliquefaciens* FZB42 + seaweed extract. Since many studies have demonstrated the importance of early root development as a critical trait determining yield formation of maize particularly with respect to P acquisition [33,34,35], special emphasis was placed on the selection of PSMs with additional root growth-promoting potential (Figure 3B).

Stabilized ammonium fertilization combined with TSP significantly improved field establishment of maize indicated by a 24% increase in shoot biomass production at 42 DAS, as compared with the unfertilized control (Table 2). This finding demonstrates that P availability was a growth-limiting factor. The importance of ammonium in this context is highlighted by the absence of growth-promoting effects in the farmer’s practice fertilization using DAP and urea without nitrification inhibitors (Table 2), leading to rapid conversion of NH_4_-N to nitrate in this treatment. However, even without additional P fertilization, the application of the PSM inoculants in combination with stabilized ammonium fully compensated the P fertilization effect of TSP and reached up to 40% increased biomass production in the Combifector-B variant, which was even larger than any plant growth-promoting effect recorded in the pot experiments under controlled conditions (Table 2, Figure 3A). The effect of Combifector-A (+ 27%) on shoot biomass production was almost identical with the result of the pot experiment (+ 28%). At the time of the intermediate harvest, no treatment differences were recorded for shoot P and N concentrations, but the N status was low [24]. The shoot P content and particularly shoot N accumulation significantly increased in response to TSP and PSM applications. This effect coincided with increased shoot biomass production (Table 2), indicating that any surplus in nutrient uptake was immediately transformed into plant growth. Similarly, shoot accumulation of micronutrients (Zn, Mn, Cu) significantly increased, particularly in the PSM treatments (Figure S1), without significant effects on the tissue concentrations, which reached the sufficiency range in all treatments [24]. The general stimulatory PSM effect on shoot accumulation of various macro- and micro-nutrients suggests root growth stimulation rather than P solubilization as a mode of action for the selected inoculants.

The improved field establishment during early growth finally translated into a significant increase in grain yield of 5.2% with TSP fertilization and of 6.5% in the FZB42 + humic acids variant, while the farmer´s practice of fertilization had the smallest yield effect (+ 3.2%) compared with the stabilized NH_4_^+^ variant without P supply (Table 2). Large effects on early field establishment may be attributed to the limited expression of adaptive responses towards improved P acquisition during the early growth of maize [4,21,33]. Localized P starter application is one of the measures to mitigate this problem [36]. Increased P availability due to ammonium-induced rhizosphere acidification in response to stabilized ammonium fertilization may induce a similar effect, followed by improved P acquisition in combination with the PSM strains with a high root growth-promoting potential, such as Combifector-A or FZB42 (Figure 3B). However, nitrification inhibitors, such as DMPP, are usually active in soils only for limited time periods of several weeks due to microbial degradation [37], and also PGPM inoculants frequently exhibit only transient effects. Therefore, no direct long-lasting effects on P solubilisation can be expected. Moreover, the initial limitations in P acquisition may be at least partially compensated, e.g., by more intensive rooting or the establishment of mycorrhizal associations in later stages of plant development [32] and the moderate P availability at the investigated field site. This could explain the limited translation of early growth effects into yield increases of only 5–6%.

## 5. Conclusions

The present study demonstrates that the expression of the plant growth-promoting and P-solubilizing potential of a wide range of bacterial and fungal PSM inoculants can be selectively influenced by the form of the N supply to the host plant with promising perspectives for synergistic effects with stabilized ammonium fertilization. The results clearly demonstrate that the beneficial effects are not necessarily related with a direct improvement of the P solubilizing potential of the PSM strains. It remains to be established to which extent root-induced rhizosphere acidification in response to ammonium uptake contributes to the expression of the effects. Increased auxin production potential of the inoculants with ammonium as the preferential N source [38] or a stimulatory effect on ammonium-induced proton extrusion of plant roots recently reported for selected *Bacillus* strains [39] as well as stimulation of rhizosphere acid phosphatase activities in response to a lower rhizosphere pH could provide additional explanations. It can also be expected that not only plant-PSM associations, but also inoculants expressing only root growth-promoting activity would profit from the combination with stabilized ammonium fertilizers. In these cases, the formation of a larger acidifying root system may contribute to the solubilisation of acid-soluble P sources (e.g., Ca-P, Rock-P, ashes, slags, etc.) as well as micronutrients (Fe, Zn, Mn, Cu) at least on soils with neutral to alkaline pH. This would not only support plant species with low inherent potential for root-induced nutrient mobilization, but also the expression of adaptive mechanisms for the solubilisation of sparingly soluble soil nutrients.

The finding that a wide range of different bacterial and fungal inoculants had beneficial effects on plant growth and/or nutrient mobilization in combination with stabilized ammonium fertilization raises the question whether native populations of PGPMs could also be influenced in a similar way. These interactions might at least partially contribute to the positive effects on nutrient acquisition and plant growth promotion observed in the non-inoculated controls supplied with stabilized ammonium fertilization. Apart from rhizosphere acidification, ammonium-dominated fertilization also significantly modifies the composition of root exudates compared with the nitrate supply [40,41] due to intense transcriptomic, proteomic, and metabolic alterations related with the assimilation of ammonium [42,43]. Accordingly, distinct rhizosphere microbiome effects can be expected. However, surprisingly, numerous studies have addressed the impact of N fertilization intensity on soil microbial communities [44,45,46], while N form effects have rarely been investigated so far [47]. These aspects need to be considered for future investigations together with the impact of different soil properties, climatic conditions, and genotypic differences in crop responsiveness to evaluate the potential of stabilized ammonium fertilizers as tools to manipulate plant interactions with plant growth-promoting microorganisms.

## 6. Patents

Some result in this article has been submitted for a joint patent application in 2017 by University of Hohenheim and Eurochem Agro GmbH to the European Patent Office (application number EC70522EP SF/IRK on “Method and Composition for Improving Nutrient Acquisition of Plants”.

## Figures and Tables

**Table 1:** Mineral nutritional status of maize (cv Colisee) grown on a clay-loam, organic farming soil (pH 7.0), supplied with and without (No P) P fertilization in the form of Rock-P or soluble triple superphosphate (TSP) and N supply in the form of Ca-nitrate (NO_3_) or DMPP-stabilized ammonium (NH_4_) as affected by different PSM inoculants (Figure 3).

	N	P	K	Mn
**Shoot Mineral Concentration (mg g^−1^)**				
No P	12.4 d	2.0 d	41.3 ab	0.02 b
NH_4__Rock-P	25.6 ab	2.5 ab	45.9 ab	0.02 b
NH_4__ Rock-P _Trianum P	35.2 ab *	2.4 abc	45.0 ab	0.03 a *
NH_4__ Rock-P _Proradix	33.9 ab *	2.3 abcd	45.9 ab	0.03 a *
NO_3__ Rock-P _Proradix	36.4 ab *	2.6 a	46.6 a	0.03 a *
NH_4__ Rock-P _Rhizovital	34.6 ab *	2.2 bcd	44.0 ab	0.03 a *
NH_4__ Rock-P _Paenibacillus	31.9 b *	2.2 bcd	42.9 ab	0.03 a *
NH_4__ Rock-P _BFOD	37.4 a *	2.3 abcd	45.5 ab	0.03 a *
NH_4__ Rock-P _Vit SP11	34.2 ab *	2.2 bcd	40.8 ab	0.03 a *
NH_4__ Rock-P _CombifectorA	34.6 ab *	2.1 cd	41.2 ab	0.03 a *
NO_3__TSP	35.0 ab *	2.2 bcd	39.5 b	0.03 a *
**Shoot Mineral Content (mg Plant^−1^)**				
No P	45.7 d	7.4 d	151.6 d	0.07 d
NH_4__Rock-P	271.5 bc	19.5 bc	356.6 bc	0.24 bc
NH_4__ Rock-P _Trianum P	311.7 ab	21.3 abc *	398.8 ab *	0.27 abc
NH_4__ Rock-P _Proradix	330.9 a *	22.2 ab *	448.9 a *	0.32 a *
NO_3__ Rock-P _Proradix	250.2 c	17.7 c	317.0 c	0.22 c
NH_4__ Rock-P _Rhizovital	328.1 a	20.9 abc	415.1 ab *	0.28 abc *
NH_4__ Rock-P _Paenibacillus	302.6 abc	21.4 abc	409.9 ab *	0.27 abc
NH_4__ Rock-P _BFOD	318.3 ab	19.6 bc	386.2 abc	0.27 abc
NH_4__ Rock-P _Vit SP11	339.9 a *	22.0 abc	404.7 ab *	0.29 abc *
NH_4__ Rock-P _CombifectorA	341.7 a *	20.4 bc	405.0 ab *	0.30 ab *
NO_3__TSP	289.0 abc	25.0 a	443.1 a	0.26 abc

Means of five replicates. One-way ANOVA, Tukey test. Different letters indicate significant differences (*p* < 0.05); * indicates significant differences after pairwise comparison of PSM-inoculated variants versus the non-inoculated control with ammonium fertilization (*t*-test, *p* < 0.05).

**Table 2:** Shoot dry matter, P and N-nutritional status during early growth (42 DAS), and final grain yield of maize (cv Limagrain 30.600) on an alkaline clay loam soil (Vertic Xerofluvent, pH 8.6) with and without (no P) P fertilization in the form of triple superphosphate (TSP) or di-ammonium phosphate (DAP). Nitrogen was supplied as DMPP-stabilized ammonium sulfate (stabilized NH_4_^+^) or non-stabilized Urea-DAP. In the PSM variants, phosphate fertilization was replaced by selected PSM products: Combifector-A (Combi-A), Combifector-B (Combi-B), *Bacillus amyloliquefaciens* FZB42 (FZB42) + humic acids (HA), *B. amyloliquefaciens* + seaweed extract. Nutrient (P, N) data refer to shoot concentrations in % and to shoot contents per plant (data in brackets). Means of four replicates. Different letters indicate significant differences. One way ANOVA (*p* < 0.05).

Treatment	Shoot DM_(42 DAS)_ (g)	Grain Yield (t ha^−1^)	Shoot-P_(42 DAS)_ % (mg plant^−1^)	Shoot-N_(42 DAS)_ % (mg plant^−1^)
Stabilized NH_4_^+^ no P	33.3 c	15.3 d	0.45 a (0.15 b)	3.3 a (1.08 c)
Stabilized NH_4_^+^ + TSP	41.2 ab (+ 24%)	16.1 ab (+ 5.2%)	0.48 a (0.20 ab)	3.2 a (1.35 ab)
Stabilized NH_4_^+^ + Combi-A	42.4 ab (+ 27%)	15.9 ab (+ 3.9%)	0.47 a (0.20 ab)	3.3 a (1.42 ab)
Stabilized NH_4_^+^ + Combi-B	46.7 a (+ 40%)	16.0 ab (+ 4.0%)	0.48 a (0.22 a)	3.4 a (1.58 a)
Stabilized NH_4_^+^ + FZB42+HA	44.4 a (+ 33%)	16.3 a (+ 6.5%)	0.47 a (0.21 a)	3.3 a (1.48 a)
Stabilized NH_4_^+^ + *B. amylolique-faciens* + seaweed extract	45.6 a (+ 37%)	15.6 bcd (+ 1.9%)	0.44 a (0.20 ab)	3.3 a (1.50 a)
Urea+DAP (farmers practice)	36.6 c (+ 10%)	15.8 abc (+ 3.2%)	0.48 a (0.18 ab)	3.2 a (1.35 ab)

## Supplementary Materials

The following figure is available online at http://www.mdpi.com/2076-2607/7/2/38/s1, Figure S1: Micronutrient shoot accumulation during early growth (42 DAS) of Maize (cv Limagrain 30.600) on an alkaline clay loam soil (Vertic Xerofluvent, pH 8.6) with and without (NoP) P fertilization in the form of triple superphosphate (TSP) or di-ammonium phosphate (DAP). Nitrogen was supplied as DMPP-stabilized ammonium sulfate or non-stabilized Urea-DAP. In the PSM variants, phosphate fertilization was replaced by selected PSM products: Combifector-A (CombiA), Combifector-B (CombiB), *Bacillus amyloliquefaciens* FZB42 + humic acids (FZBHA), B. amylolique-faciens + seaweed extract (BaSE). Means of four replicates. Different letters indicate significant differences; One way ANOVA (*p* < 0.05). Table S1: Effects of nitrate (NO_3_) versus stabilized ammonium (Stab. NH_4_) fertilization on shoot growth and yield formation of different crops with and without inoculation with microbial biostimulants (BS).
