# Erratum: Mpanga, I.K.; et al. The Form of N Supply Determines Plant Growth Promotion by P-Solubilizing Microorganisms in Maize. *Microorganisms* 2019, *7*, 38

**DOI:** 10.3390/microorganisms7040111

**Published:** 2019-04-25

**Authors:** Microorganisms Editorial Office

**Affiliations:** MDPI, St. Alban-Anlage 66, 4052 Basel, Switzerland; microorganisms@mdpi.com

The following changes have been made to the published paper [1]. Figure 3B was missing, while Figure 3A showed twice in the previously published version.

Please find the correct Figure 3 below:

In summary, these changes did not impact in any way the significance of the overall results or the conclusions of our paper. We updated the manuscript, and the original version will remain online. We apologize for any inconvenience we may have caused to our readers.

## Figures and Tables

**Figure 3:**
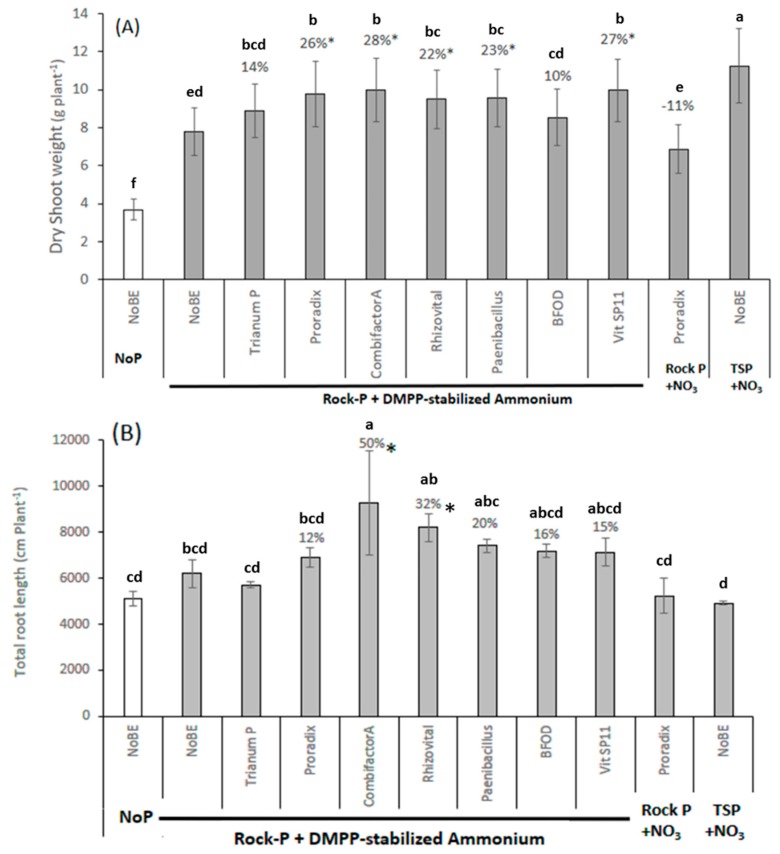
Shoot biomass (**A**) and total root length (**B**) of maize (cv Colisee) grown on a clay-loam, organic farming soil (pH 7.0), supplied with and without (No P) P fertilization in form of Rock-P or soluble triple superphosphate (TSP). N supply in the form of Ca-nitrate (NO_3_) or DMPP-stabilized ammonium. Microbial inoculants: *Trichoderma harzianum* T22 (Trianum P), *Pseudomonas* sp. DSMZ 13134 (Proradix), *Trichoderma harzianum* OMG16 + 5 *Bacillus* strains (Combifector-A); *Bacillus amyloliquefaciens* FZB42 (Rhizovital), *Paenibacillus mucilaginosus*, *Penicillium* sp. PK 112 (BFOD), Vitalin SP11 (VitSP11), or no inoculation (NoBE). Means of five replicates. One-way ANOVA, Tukey test. Different letters indicate significant differences (*P* < 0.05); * indicates significant differences after pairwise comparison of PSM-inoculated variants versus the non-inoculated control with ammonium fertilization (*t*-test, *P* < 0.05).
