# Supplementary material for: The Form of N Supply Determines Plant Growth Promotion by P-Solubilizing Microorganisms in Maize
Source: Microorganisms. 2019 Jan 29;7(2):38. doi: 10.3390/microorganisms7020038 (PMC6406690; doi:10.3390/microorganisms7020038)
Supplement: Supplementary file 1 [file microorganisms-07-00038-s001.pdf]

Table 1. Effects of nitrate (NO_3) versus stabilized ammonium (Stab. NH_4) fertilization on shoot growth and yield formation of different crops with and without inoculation with microbial biostimulants (BS).

Microbial Biostimulant (BS)	Soil/pH/P source	NO_3	NO_3+BS	Stab. NH_4	Stab. NH_4+BS	Crop, Culture system	Growth parameter	Reference
Proradix	pH 7.6 Rock-P (Exp.2, limed)	9.3 c	14.5 b	16.9 b	20.4 a	Maize	Shoot FW (g)	[1]
Proradix	Silty loam,	4.3 b	4.6 b	8.1 a	9.6 a	Maize (Pot)	Shoot DM (g)	[2]
FZB42	pH 7.0 Rock-P	1.8 c	2.0 bc	2.9 b	4.1 a	Maize (Pot)	Shoot DM (g)	[3]
Combi-B	(Exp.3)	2.0 b	3.2 b	4.1 ab	5.8 a	Maize (Pot)	Shoot DM (g)	[4]
Proradix	Clay-loam, pH 6.8, Rock-P		10.4 c	13.0 b	15.4 a	Maize (Pot)	Shoot DM	[1]
Proradix	Silty loam,	12.5 b	13.5 b	11.4 b	15.4 a	Wheat (Pot)	Grain DM	[1]
P. mucilaginosus	pH 6.4 Rock-P	12.5 b		11.4 b	15.1 a		Grain DM	[1]
FZB42	Silty sand, pH 5.6 Rock-P			7.3 b	10.0 a	Tomato (Pot)	Shoot DM	[5]
CRENEL	Silty loam, pH 5.7 starter P	2.1 b	2.3 b	2.6 b	3.4 a	Maize (Pot)	Shoot DM	[6]
CRENEL	95% Sand,			300 b	640 a	Tomato (field)	Shoot FW	[7]
	pH 7.9 No P			17.2 b	35.8 a		Yield (t ha^{-1})	

Proradix = *Pseudomonas* sp. DMSZ13134 (Sourcon Padena, Tübingen, Germany); FZB42 = *Bacillus amyloliquerfaciens* FZB42; *Paenibacillus* mucilaginosus (ABITEP, Berlin Germany), CRENEL = Microbial Consortia Product (Eurochem-Agro., Mannheim, Germany); Combi-B: FZB42-*Trichoderma harzianum* OMG16 combination with Zn and Mn (Institute of Bioanalytical Sciences, Bernburg, Germany).

Reference

1. Nkebiwe P. M. Fertilizer placement and the potential for its combination with bio-effectors to improve crop nutrient acquisition and yield. Dissertation, Universität Hohenheim, Stuttgart, Germany, 20 December 2017.
2. Gomez-Genao N.J. Factors determining N-form dependent efficiency of microbial bio-effectors in maize. Master Thesis, Universität Hohenheim, Stuttgart, Germany, 2017.
3. Mpanga, I.K. Unpublished work. Universität Hohenheim, Stuttgart, Germany.
4. Hsu C.T. Soil type-dependent expression of PGPR effects in maize as affected by the form of nitrogen fertilization. Master Thesis, Universität Hohenheim, Stuttgart, Germany, 2018.
5. Mpanga, I.K.; Dapaah, H.K.; Geistlinger, J.; Ludewig, U.; Neumann, G. Soil Type-Dependent Interactions of P-Solubilizing Microorganisms with Organic and Inorganic Fertilizers Mediate Plant Growth Promotion in Tomato. *Agronomy* **2018**, *8*, 213.
6. Sittinger M. Effects of a microbial consortia product on the growth of maize (Zea mays). Master Thesis, Universität Hohenheim, Stuttgart, Germany, 2018.
7. Bradacova K. et al. Microbial Consortia versus Single-Strain Inoculants: an Advantage in PGPR-assisted Tomato Production? *Agronomy*, submitted.

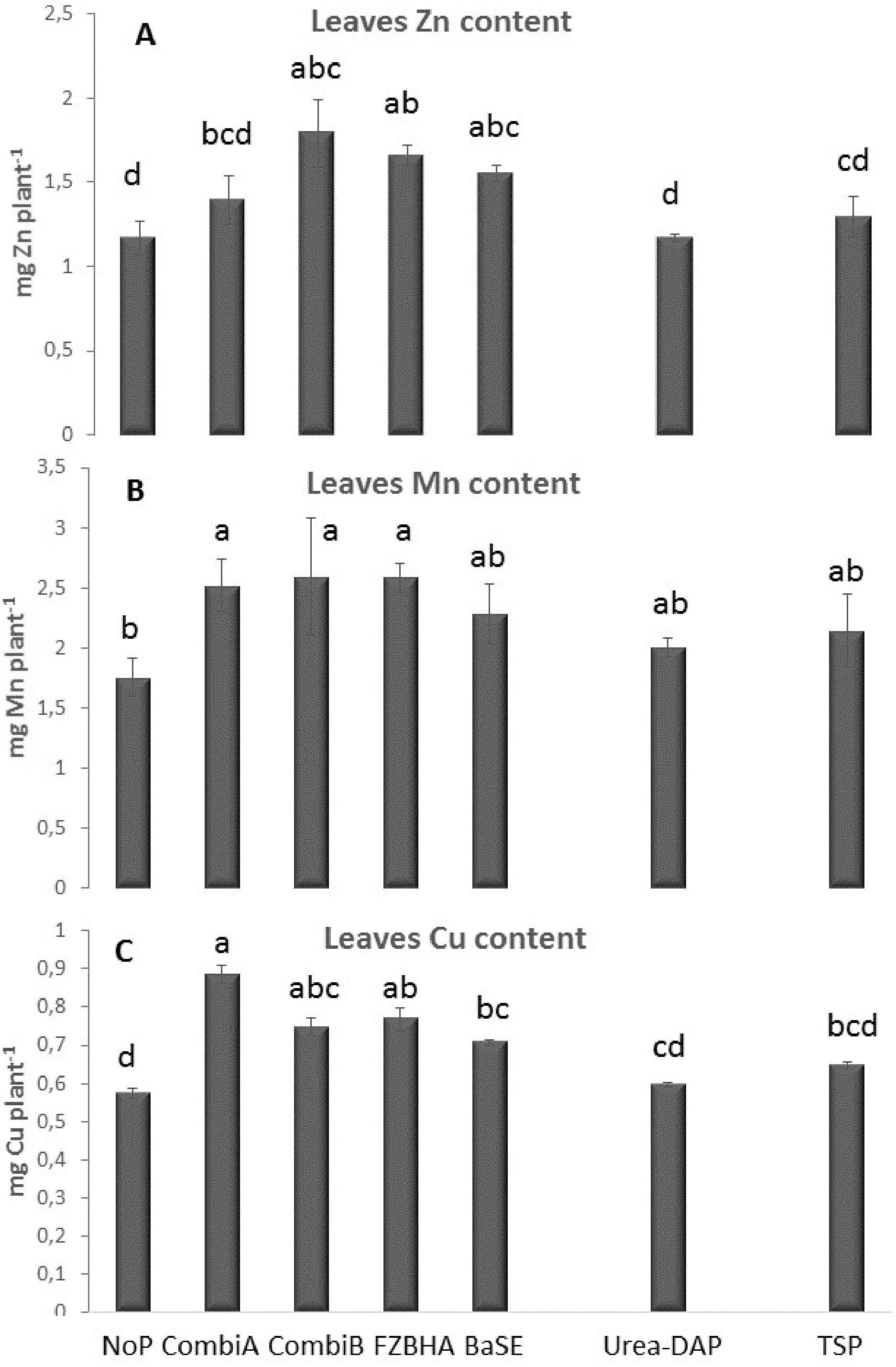

Figure S1. Micronutrient shoot accumulation during early growth (42 DAS) of Maize (cv Limagrain 30.600) on an alkaline clay loam soil (Vertic Xerofluvent, pH 8.6) with and without (NoP) P fertilization in form of triple superphosphate (TSP) or di-ammonium phosphate (DAP). Nitrogen was supplied as DMPP-stabilized ammonium sulfate or non-stabilized Urea-DAP. In the PSM variants, phosphate fertilization was replaced by selected PSM products: Combifector-A (CombiA), Combifector-B (CombiB), *Bacillus amyloliquefaciens* FZB42 + humic acids (FZBHA), *B. amylolique-faciens* + seaweed extract (BaSE). Means of four replicates. Different letters indicate significant differences; One way ANOVA ($p < 0.05$).
